# Simultaneous Analysis of 20 Mycotoxins in Grapes and Wines from Hexi Corridor Region (China): Based on a QuEChERS–UHPLC–MS/MS Method

**DOI:** 10.3390/molecules23081926

**Published:** 2018-08-02

**Authors:** Bo Zhang, Xia Chen, Shun-Yu Han, Min Li, Teng-Zhen Ma, Wen-Jun Sheng, Xia Zhu

**Affiliations:** Gansu Key Laboratory of Viticulture and Enology, College of Food Science and Engineering, Gansu Agricultural University, Lanzhou 730070, China; zhangbo@gsau.edu.cn (B.Z.); chenxia8874@163.com (X.C.); limin3302@163.com (M.L.); matz@gsau.edu.cn (T.-Z.M.); shengwenjun@gsau.edu.cn (W.-J.S.); zhux@gsau.edu.cn (X.Z.)

**Keywords:** QuEChERS, UHPLC–MS/MS, mycotoxins, grapes and wines, hexi corridor region

## Abstract

The aim of this study is to develop and validate an improved analytical method for the simultaneous quantification of 20 types of mycotoxins in grapes and wines. In this research, the optimization of tandem mass spectrometer (MS/MS) parameter, ultra-high pressure liquid chromatography (UHPLC) separation, and QuEChERS procedure, which includes wine/water ratio, the amount and type of salt, clean-up sorbent, were performed, and the whole separation of mycotoxins was accomplished within 7 min analyzing time. Under optimum conditions, recoveries ranged from 85.6% to 117.8%, while relative standard deviation (RSD) remained between 6.0% and 17.5%. The limit of detection (LOD, 0.06–10 μg/L) and the limit of quantification (LOQ, 0.18–30 μg/L) were lower than those permitted by legislation in food matrices, which demonstrated the high sensitivity and applicability of this efficient method. Finally, 36 grapes and 42 wine samples from the Hexi Corridor region were analyzed. Penicillic acid (PCA), mycophenolic acid (MPA), cyclopiazonic acid (CPA), fumonisin B1 (FB1) and zearalenone (ZEN) were detected in a small number of grape samples with lower concentrations between 0.10 μg/L and 81.26 μg/L. Meanwhile, ochratoxin A (OTA), aflatoxin B2 (AFB2), MPA, CPA, and ZEN were detected in some wine counterparts with concentrations ranged from 0.10 μg/L to 4.62 μg/L. However, the concentrations of the detected mycotoxins were much lower than the maximum legal limits set of other products.

## 1. Introduction

Mycotoxins are fungal secondary metabolites. Due to their chemical properties and concentration levels, mycotoxins have been considered as the cause of acute and chronic diseases of both human beings and animals. Moreover, mycotoxins and their toxic transformation products might survive from the process of the foodstuff production and completely remain active in the final product, which can aggravate the same problem again [1].

Grapes are vulnerable to be affected by various types of fungal, and the toxinogenic fungi can produce mycotoxins [2]. Previous studies indicated that there was a correlation between physiochemical qualities of wine grapes and the presence of all isolated fungi [3]. Although sophisticated treatment has been applied in the process of grape planting and wine making, fungal contamination is still unavoidable. In addition, the climate impact and the excessive application of nitrogen fertilizer would also encourage the spread and breeding of fungi [4]. Therefore, the safe quality of grapes and wines should be seriously considered, in order to avoid mycotoxin contaminations. 

Nowadays, hundreds of mycotoxins with vivid structures have been identified, and their corresponding physical and chemical properties have been explored in-depth [5]. Meanwhile, more than 99 countries have adopted regular monitoring and surveillance of mycotoxins in foods [6]. Since the first report of ochratoxin A (OTA) in wine in 1995 [7], a range of data have been generated from research regarding wines, grape juices and raisins. For example, Logrieco and his coworkers identified the fumonisin B2 (FB2) in grapes and Italian red wines from 2009 to 2010 [8,9]. Thereafter, 77 wine samples from 13 countries were tested and, unfortunately, 18 of them had been contaminated with FB2 in the range from 1 mg/L to 25 mg/L [10]. Similar results were identified in wine samples from Spain [11]. Moreover, the OTA, alternariol (AOH), cyclopiazonic acid (CPA), mycophenolic acid (MPA) and zearalenone (ZEN) were also found in wine samples from the Dutch market [12]. Thus, the occurrence of mycotoxins in wines has been widespread.

A large number of analytical methods have been employed to control mycotoxins in foods, such as enzyme-linked immunosorbent assay (ELISA) [13], thin-layer chromatography (TLC) [14], gas chromatography–mass spectrometer (GC–MS) [15], and high-performance liquid chromatography (HPLC) [16]. However, these methods are only valid to one specific or a small amount of mycotoxins. Recently, with the development of chromatographic and mass spectroscopic instruments, ultra-high pressure liquid chromatography–tandem mass spectrometry (UHPLC–MS/MS) technologies have been proposed for multiclass mycotoxins analysis, due to their superior sensitivity, specificity and efficiency.

Additionally, in order to minimize the loss of target analytes and subsequent decrease of recoveries, a lot of effort has been made to develop and standardize techniques of sample preparation. Many extraction procedures of liquid samples have been described in literature, such as wines, beers, juices and milks, based on solid-phase extraction (SPE) through the application of immunoaffinity columns (IAC) or other types of sorbents [12]. The other extraction methods such as liquid–liquid extraction (LLE) [17], solid-phase microextraction (SPME) [18], stir bar sorptive extraction (SBSE) [19], and liquid-phase microextraction (LPME) [20] have also been applied. Nowadays, an efficient and affordable method has been widely applied due to its features of quick, easy, cheap, effective, rugged and safe, which is therefore called QuEChERS. This extraction technology is reliable with a number of advantages, such as simplicity, minimum steps, and effectiveness in cleaning-up complex samples. Liu, et al. [21] reported a modified QuEChERS method combined with UHPLC–MS/MS for the simultaneous determination of 26 mycotoxins in sesame butter, which enabled the detection of the multiclass mycotoxins at limit of quantitation to be significantly lower than the standard residue levels regulated by the European Union (EU) regulations. 

Hexi Corridor region (Gansu Province, Northwest China) is the historic and the major production area of wine in China (Figure S1, Supplementary Material). The year-round dry and cold climate in this region (Table S1) guarantees fewer pest and disease problems to the grape planting. Therefore, it has also been recognized as the major production area of organic grapes and wines in China. In order to improve the overall quality of local wines, it is essential to monitor the basic safety indexes of grape materials and wine productions. In this study, a modified QuEChERS for the extraction of 20 mycotoxins in grapes and wines was proposed. Additionally, UHPLC–MS/MS was selected as the preferred detection method. Finally, the validated and optimized method was successfully applied in the investigation of mycotoxins in regularly analyzed grape and wine samples in Hexi Corridor region. To our knowledge, this study can be the first report regarding the application of QuEChERS–UHPLC–MS/MS to the analysis of mycotoxins in grapes and wines in this area.

## 2. Results and Discussions

### 2.1. Optimization of MS/MS Parameters and Chromatographic Separation

To obtain the simple and effective method for the extraction and analysis of major mycotoxins in grapes and wines, a range of factors that might influence the efficiency of the method were carefully investigated. The 20 mycotoxins were injected directly into the MS/MS system and analyzed through the MS full scan. The result showed that most analytes can be better ionized in the positive mode with the presentation of abundant [M + H]^+^ ion, while the ammonium adducts [M + NH_4_]^+^ were selected for the detection of the diacetoxyscirpenol (DAS), neosolaniol (NEO) and T-2 toxin (T-2). It is also demonstrated by the results that HT-2 toxin (HT-2) can be ionized through the application of [M + Na]^+^ adduct (Table 1). Since the precursor ions were determined, different collision energies were applied to obtain product ions for each mycotoxin. The top 2 most intense product ions generated from each precursor ion were selected as the quantifier and qualifier ions which can comply with the criteria and guidelines for confirmatory analysis (Table 1).

**Figure 1:**
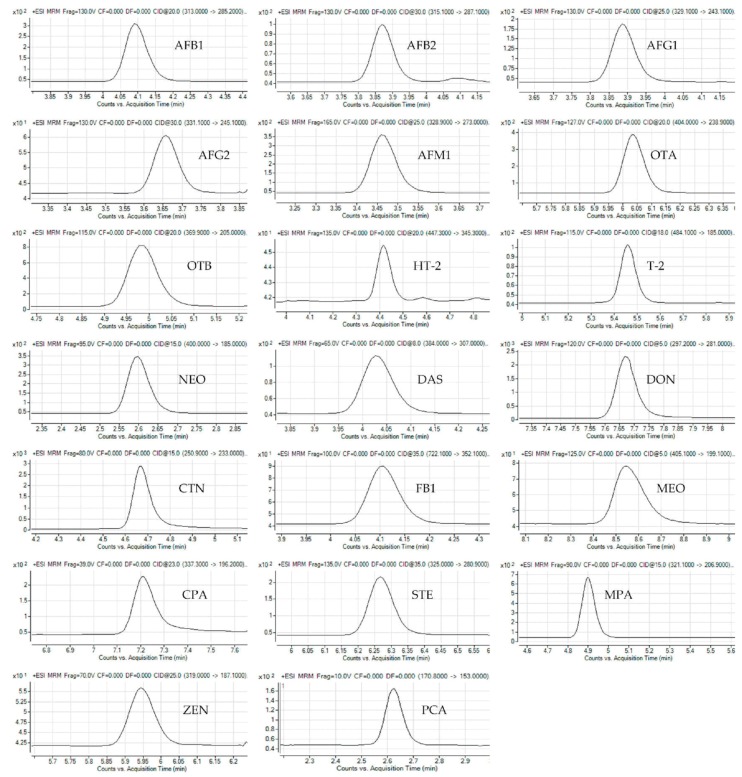
The extracted ion chromatograms of 20 mycotoxins in a standard solution under positive mode.

In order to improve the separation efficiency of mycotoxins, effects of chromatographic solvents, which are composed of methanol–water, methanol–water (0.1% formic acid), acetonitrile–water, acetonitrile–water (0.1% formic acid) and acetonitrile (0.1% formic acid) –water (0.1% formic acid) respectively, were compared. It can be seen from the results that the acetonitrile can be a preferred organic modifier to overcome peak splitting and tailing (data not shown). The acetonitrile can also dilute the matrix in the area of chromatogram, in which analytes of interest were eluted. This means that acetonitrile would be the best choice. Similar results were also reported by Zhao et al. [22], who found that acetonitrile can provide higher extraction recovery for most of the target analytes, compared with other organic solvents (acetone). Particularly, when formic acid (0.1%) was added to acetonitrile solvent system, all peaks would demonstrate the good shape and high sensitivity (Figure 1). Consequently, the optimized positive electrospray ionization mode (ESI^+^) parameters for the mycotoxins, obtained by direct infusion of the analyte solutions diluted in acetonitrile–water with 0.1% formic acid, were applied for the following experiment.

### 2.2. Optimization of the QuEChERS Procedure

#### 2.2.1. Selection of Wine/Water Ratio

An appropriate wine/water ratio can increase the sensitivity of the analytes determination. As shown in Table 2, the best compromise, which can be achieved by the 5:5 wine/water ratio, was employed. On the other hand, a significant matrix effect of several compounds [when aflatoxin M1 (AFM1), fumonisin B1 (FB1) were severely suppressed, and when DAS and T-2 were severely enhanced] under arbitrary wine/water ratio was recorded, when the ion suppression or enhancement was obvious.

#### 2.2.2. Selection of Salt Amount and Type

The salting-out effect is a significant parameter for the QuEChERS procedure. Generally, the addition of salt into aqueous phase can increase the ionic strength, which facilitates the transformation of the analytes from aqueous phase to organic phase [22]. In this study, MgSO_4_ and NaCl were considered as two candidates to be tested. It should be noted that the extraction efficiency of most mycotoxins can enhance with the increase in MgSO_4_ (Table 3). However, when the amount of MgSO_4_ increased to over 4.0 g, the heating and agglomeration of the whole system was apparent, and AFM1 and sterigmatocystine (STE) could hardly obtain the requirements of recovery. To overcome this deficiency, NaCl was selected to be combined with MgSO_4_, which is less exothermic than MgSO_4_ [23]. In this process, the recoveries of mycotoxins, such as citrinin (CTN), ochratoxin B (OTB), AFM1, DAS, NEO, OTA, STE and T-2, increased significantly when the additive amount of NaCl was fixed at 1.0 g, while the additive amount of MgSO_4_ was increased gradually. A possible reason was that the NaCl enabled the separation of acetonitrile from aqueous phase to be more thorough [24]. Furthermore, due to the stronger drying characteristics of MgSO_4_, the greater the amount of MgSO_4_ added, the stronger the water absorption, and the higher the extraction efficiency of the analytes could be achieved. Based on this result, the combination of 1.0 g NaCl and 4.0 g MgSO_4_ was used in further steps.

#### 2.2.3. Selection of Clean-Up

In this experiment, several types of dispersive SPE (dSPE) sorbents were studied, which include C18, PSA, GCB and sorbent combinations (C18 + GCB, C18 + PSA, PSA + GCB). Previous studies found that C18 and GCB can remove nonpolar matrix components, such as pigments and sterols, while PSA can absorb polar counterparts and effectively eliminate sugars, fatty acids and organic acids [25]. As presented in Table S2, it can be demonstrated that C18 adsorbent can achieve satisfactory recoveries of 67–121% for all mycotoxins, while the GCB can absorb almost all the mycotoxins with planar structure, such as OTA, AFM1, aflatoxin G2 (AFG2) and STE, and dramatically reduce their corresponding recoveries by 23%, 18%, 13% and 3%, respectively. The addition of PSA which can lead to the recoveries of OTB was about 30%, and FB1 was almost totally lost. The combination of C18 + GCB and PSA + GCB can hardly be practical for the clean-up step, since a large amount of mycotoxins had been lost. After using the combination of C18 + PSA, the recoveries were all satisfied apart from that of FB1. Nevertheless, when the dSPE purification step was omitted, the recoveries of the majority of mycotoxins can remain in the acceptable range (66–106%). Therefore, for the economical reason and the simplicity of the operation, no clean-up was used for further experiments, but the extra drying step will still be applied.

### 2.3. Method Validation

Validation was performed according to EU Commission Regulation (EC) No. 401/2006 and the parameters were also taken into account to ensure the reliability of the results. 

#### 2.3.1. Matrix Effect Evaluation 

Matrix effect is generally regarded as a signal suppression or enhancement of the analyte, due to the co-extracted compounds which can interact with analytes in the electrospray ionization process [26]. It could be calculated as the average percent suppression or enhancement in the peak area through the application of the following equation:

(1) $$\;\mathrm{Matrix}\;\mathrm{effect}\%\;=\;\frac{\mathrm{Peak}\;\mathrm{area}\;\mathrm{of}\;\mathrm{matrix}\;\mathrm{matched}\;\mathrm{standard}\;-\;\mathrm{Peak}\;\mathrm{area}\;\mathrm{of}\;\mathrm{solvent}\;\mathrm{standard}}{\mathrm{Peak}\;\mathrm{area}\;\mathrm{of}\;\mathrm{matrix}\;\mathrm{matched}\;\mathrm{standard}\;}\;\times \;100\;$$

**Figure 2:**
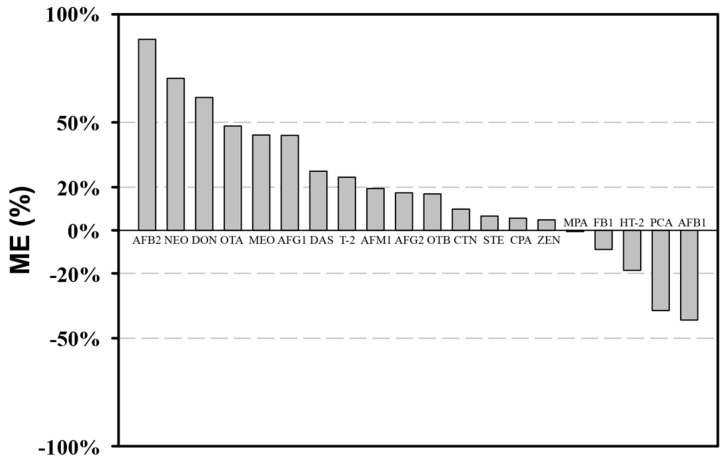
Values of matrix effect (ME) observed at 2.5 μg/L level.

In general, matrix effects can be classified into soft (suppression or enhancement of 0–20%), medium (suppression or enhancement of 20–50%), and high (suppression or enhancement >50%) matrix effects [27]. Herein, relevant results of matrix effect at 2.5 μg/L level were demonstrated in Figure 2, and 5 μg/L and 10 μg/L levels were shown in Table S3. Ten (50%) analytes showed soft matrix effects, which is in accordance to a signal suppression or enhancement equivalent to or lower than 20%. These values are adequate to generate accurate quantitative data when matrix-matched standard calibration curves are used. On the other hand, no higher matrix effects in terms of signal suppression were noted; however, the signal suppressions were medium for 2 (10%) out of 20 test analytes. Similar medium signal enhancement was observed for 5 (25%) analytes, while 3 (15%) analytes showed strong signal enhancement. In the wine solution system, the main co-extracted compounds are fatty acids, esters, alcohols and sugars, which can decrease or increase the response of the detector to the analyte [12]. In terms of most mycotoxins, when the same pretreatment was applied to grape samples, the matrix effect was still significant (>±20%), but slightly lower than that of wines (data not shown), which is similar to that of the wine matrix. Although a clean-up step was included, matrix effect remained high to most of the measured compounds. Moreover, chromatographic conditions, mass spectrometric instrumentation and ionization conditions can also influence the extent and nature of matrix effects [28]. Therefore, in order to achieve effective quantification of tested samples, matrix-matched calibration curves were used.

#### 2.3.2. Calibration Curves, Linearity, LOD and LOQ

As can be seen from Table 4, good linear relationships were achieved when correlation coefficient (*R^2^*) was greater than 0.99 in studies of range 1.25–50 μg/L to range 50–200 μg/L respectively. The analytical limits were shown based on LODs and LOQs for each mycotoxin, which were calculated in accordance to the signal-to-noise ratio approach and were found as acceptable. As also demonstrated by Table 4, the LODs in the present study were between 0.06 μg/L and 0.25 μg/L for aflatoxin B1 (AFB1), aflatoxin B2 (AFB2), aflatoxin G1 (AFG1), mevinolin (MEO), AFG2, AFM1, CPA, CTN, OTA, DAS, OTB, MPA, NEO, STE and T-2, while for the rest of the compounds, they were between 1 μg/L and 10 μg/L. Compared with previously published methods [29,30], the obtained LOQs were comparable or even more sensitive. Besides, no interfering peaks were observed at the elution zone of each analyte, which can demonstrate the excellent feasibility of this method.

#### 2.3.3. Precision and Recovery

Precision was determined by analyzing wine samples which contain all mycotoxins at concentration levels between 2.5 μg/L and 10 μg/L, and calculating the concentrations from external calibration. Data in Table S4 can show that recoveries ranging from 86% to 118% of all tested mycotoxins with RSD are lower than 20%, which is highly consistent to EU Commission Regulation (EC) No. 401/2006 (European Commission, 2006) performance criteria for quantitative methods of mycotoxins analysis. The results can demonstrate that the method presented in this study is highly accurate and reliable.

### 2.4. Application to Real Sample

Using the developed method, 78 samples, including 36 grapes and 42 wines from different vineyards and wineries in 3 geographic areas (Jiayuguan, Zhangye and Wuwei) of Hexi Corridor region, were tested. As shown in Table 5, PCA, MPA, CPA, FB1 and ZEN were detected in grape samples. In the 36 tested samples, 8 of them were contaminated with CPA ranging from <LOQ to 7.44 μg/L, with the highest frequency of occurrence 22%, while 5 of them contained MPA with total concentrations in the range of <LOQ to 81.26 μg/L, and a slightly lower frequency 14%. In addition, 2 samples were detected with PCA and ZEN at concentrations of 9.04–11.71 μg/L and 0.29–0.36 μg/L respectively. One sample was contaminated with FB1. The contamination data from the 3 different areas were also compared. The frequency of occurrence for MPA was 3%, 6% and 6% for samples obtained from Jiayuguan, Zhangye and Wuwei areas, respectively. CPA was detected in all other areas excluding Zhangye. FB1 and ZEN were found only in Wuwei, while PCA was detected in Zhangye and Wuwei areas.

In terms of the wine samples, the results showed that only 2 samples out of 42 contained OTA and AFB2 at levels higher than the LOD, and contamination levels of these two samples were 1.27 μg/L and 0.10 μg/L, respectively (Table 6). 3 samples were detected with ZEN (<LOQ to 1.85 μg/L) and 5 samples were detected with CPA (<LOQ to 4.62 μg/L), which is slightly more frequent (7% and 12% according to the analysis, respectively). In these contaminated samples, MPA had the highest frequency of occurrence (48%), with concentrations ranging from <LOQ to 2.31 μg/L. Other mycotoxins had not been detected in wines. In the detected mycotoxins, the amount of OTA did not exceed the maximum limit of 2 μg/L regulated by the EU. The concentrations of the other mycotoxins are also lower than the maximum limits set for other products. That is to say, the pollution risks of mycotoxins to grapes and wines in Hexi Corridor region are relatively low.

According to the above results, MPA, CPA and ZEN were common contaminants to both grape and wine samples. MPA and its derivatives, such as immunosuppressants, can lead to some adverse reactions and induce tumorigenesis. CPA and ZEN were neurotoxin- and estrogen-like mycotoxins, and would also exert adverse effects on the human body. Nowadays, the EU has only limited the quantity of OTA in wines and regulated its content in grapes; that is to say, OTA in wines and other grape juices for the production of drinks should be lower than 2 μg/L. However, with the increasing consumption of wine and international trade, impacts of other mycotoxins on human health should be also attached great importance by both developed and developing countries. 

## 3. Materials and Methods 

### 3.1. Chemicals and Standards

Acetonitrile, methanol and formic acid were HPLC grade (Merck, Darmstadt, Germany). Acetic acid (HPLC grade) was purchased from Dengfeng Chemical Co., Ltd. (Tianjin, China). Distilled water was obtained from Watsons (Guangzhou, China). Anhydrous magnesium sulfate (MgSO_4_) and sodium chloride (NaCl) in powder (≥99% purity) were from Dengfeng Chemical Co., Ltd. (Tianjin, China) and Damao Chemical Reagent Factory (Tianjin, China), respectively. 

The mycotoxin reference standards of AFB1, AFB2, AFG1, AFG2, CPA, MPA, OTA, PCA were supplied by Sigma-Aldrich (St. Louis, MO, USA). Standards of AFM1, CTN, DAS, DON, FB1, HT-2, NEO, OTB, STE, T-2 and ZEN were obtained from J & K Scientific Ltd. (Beijing, China). MEO was supplied by Aladdin Bio-Chem Technology Co., Ltd. (Shanghai, China).

All standards were individual stock solutions in acetonitrile or acetonitrile/water (1:1, *v/v*), and had the following concentrations: AFB2 and AFG2, 0.5 mg/L; AFB1 and AFG1, 2 mg/L; AFM1, 10 mg/L; OTA and STE, 40 mg/L; CPA, CTN, DAS, DON, FB1, HT-2, NEO, OTB, PCA and T-2, 100 mg/L; ZEN, MPA and MEO, 500 mg/L. The concentrations of mixture stock solutions were as follows: AFB2 and AFG2, 125 μg/L; AFB1, AFG1, AFM1, CPA, CTN, DAS, DON, FB1, HT-2, MEO, MPA, NEO, OTA, OTB, PCA, STE, T-2 and ZEN, 500 μg/L. The individual and mixture stock solutions were stored at −20 °C. 

### 3.2. Optimization of the Extraction Method

#### 3.2.1. The Wine/Water Ratio Optimization

In order to reduce interference from the matrix compounds and solve problems of phase separation during the extraction process, 6 types of wine/water ratios were selected, including 1:9, 2:8, 3:7, 5:5, 7:3 and 8:2 (*v/v*). Recoveries of the test analytes corresponding to different wine/water ratios were estimated using solvent-based calibrations and compared.

#### 3.2.2. The Amount and Type of Salt Optimization

Under the optimal wine/water ratio determined above, the sole additive amount of MgSO_4_ and the combined additive amount of MgSO_4_ and NaCl were optimized. 1.0–4.0 g MgSO_4_ was respectively chosen to test. Meanwhile, taking into consideration the serious heating and agglomeration phenomena in the case of large additive amount of MgSO_4_, which could result in the loss of some mycotoxins, the combined addition of MgSO_4_ and NaCl was tested. The combinations of salt including (I) 1.0 g NaCl, 1.0 g MgSO_4_; (II) 1.0 g NaCl, 2.0 g MgSO_4_; (III) 1.0 g NaCl, 3.0 g MgSO_4_; (IV) 1.0 g NaCl, 4.0 g MgSO_4_ were selected successively and added in the sequence of first NaCl and then MgSO_4_. The recoveries of 20 kinds of mycotoxins were used as indexes to determine the optimal added salt amount and salt types by a series of experiments.

#### 3.2.3. The Clean-Up Optimization

In order to improve recoveries provided by the aforementioned procedure, various dSPE sorbents (C18, PSA, GCB; Sigma-Aldrich, St. Louis, MO, USA) and sorbent mixtures (C18 + GCB, C18 + PSA, PSA + GCB) were tested during the clean-up step, respectively. 

#### 3.2.4. Optimized QuEChERS Procedure

Lastly, the optimized QuEChERS extraction procedure was employed to extract target analytes from the examined matrices. Five grams of homogenized sample were weighed into a 50 mL polypropylene centrifuge tube, 5.0 g distilled water and 10 mL acetonitrile (containing 1% acetic acid) were added. Vigorous shaking (3000 rpm) of the mixture (1 min) at room temperature was followed by the addition of 1.0 g NaCl and 4.0 g MgSO_4_. Then, the sample was shaken for 1 min and centrifuged again for 5 min (13,000 rpm) at 10 °C. And 3 mL aliquot of the upper organic phase was transferred into a 10 mL polypropylene tube containing 450 mg MgSO_4_, further shaking for 30 s and centrifugation (5 min, 5000 rpm, 10 °C). Afterwards, approximate 0.5 mL extract were taken for analysis. Prior to UHPLC–MS/MS measurements, the extract was passed through a 0.22 μm filter (Millipore, Bedford, MA, USA) and diluted with 0.5 mL of methanol.

### 3.3. Chromatographic and Mass Spectrometric Conditions

An Agilent 1290 series UHPLC system coupled to a 6460 Triple Quadrupole (QqQ) mass spectrometer (both Agilent Technologies, Waldbronn, Germany) was used to analyze the samples. Precursor and product ion selection as well as the optimization of collision energies were performed with flow injection of single-analyte solutions. UHPLC separations were performed in a reversed-phase C18 analytical column of 50 mm × 2.1 mm and 1.8 μm particle size (ZORBAX RRHD Eclipse Plus C18) by Agilent Technologies (Waldbronn, Germany).

The chromatographic solvents were water 0.1% formic acid solution (eluent A) and acetonitrile 0.1% formic acid (eluent B). The gradient program was as follows: 0.0 min, 10% B; 2.4 min, 42% B; 6.0 min, 51% B; 6.2 min, 95% B; 7.0 min, 10% B. A subsequent re-equilibration time (5 min) should be performed before next injection. The constant flow rate was 0.3 mL/min while the injection volume was 2 μL. Moreover, the column temperature was maintained at 30 °C.

MS/MS analyses of mycotoxins were performed on an 6460 QqQ mass spectrometer with Agilent Jet Stream Technology under the dynamic multiple reaction monitoring (DMRM) conditions in ESI^+^. The following settings were used: nebulizer, 40 psi; drying gas temperature, 350 °C; drying gas flow, 10 L/min; capillary voltage, 4000 V.

### 3.4. Method Validation Study

Performance characteristics of the optimized method were established by a validation procedure with samples, studying linearity, LODs and LOQs, accuracy, precision and matrix effect.

For the linearity test and for the determination of LODs and LOQs, working standard solutions were prepared at 6 concentration levels within the range from 1.25 μg/L to 50 μg/L for AFB2 and AFG2; from 5 μg/L to 200 μg/L for the rest of mycotoxins (*n* = 6). Calculation curves were performed based on the average peak areas by external standard calibration. RSD for each individual calibration level, calibration curve regression equations with their *R*^2^ were calculated and the linear range for each mycotoxin was determined. LODs and LOQs were calculated as produce chromatographic peak at signal-to-noise ratio (S/N) of 3 and 10, respectively. The accuracy and precision of the method were evaluated through recovery experiments by spiking mycotoxins to a blank aliquot of wine (wine without mycotoxins). The analysis of the samples was performed in 6 replicates. Furthermore, the matrix effect was evaluated according to the previous research [31].

### 3.5. Analysis of Mycotoxins in Grape and Wine Samples

The validated method was used to detect the presence and quantify of the target mycotoxins in grape and wine samples from 3 different geographic areas of Hexi Corridor Region, which are Jiayuguan, Zhangye and Wuwei, respectively (Figure S1). There are 36 grape samples in total, collected in 2017 immediately after the harvest from 7 vineyards. Forty-two wine samples, which include 6 commercial brands, were selected during the years 2007–2016 from various wineries. At least 3 kg or L/sample was collected, and all samples were stored in the dark at 4 °C before analysis.

### 3.6. Data Analysis

Mycotoxin identification and quantitation analyses in grape and wine samples were performed using Agilent’s Mass Hunter Quantitative Analysis Software (version B.07.00). Microsoft Excel 2007 (Microsoft, Redmond, WA, USA) was used to calculate the means, standard deviations and relative standard deviations.

## 4. Conclusions

Mycotoxins are secondary fungi metabolites present in foods which can cause adverse effects on humans and animals. Therefore, it is essential to develop a simple, effective, sensitive and validated analytical method to monitor mycotoxins. In this work, a fast and simple analytical method was developed and optimized for the analysis of 20 types of mycotoxins in grapes and wines, based on a formic acidified acetonitrile–water extraction, followed by a partitioning and a subsequent drying step with NaCl and MgSO_4_. The clean-up step by dSPE is left out. The samples, after treated by extra MgSO_4_, can be injected-tested by UHPLC–MS/MS with ESI^+^ mode, which avoids the use of expensive and complicated IAC operation and the concentration process. The results obtained in the validation procedure showed satisfactory validation parameters in terms of linearity, accuracy, precision, selectivity, LODs and LOQs. The linearity range was sufficient within the tested concentration range, with *R*^2^ higher than 0.99 in all cases. Finally, the 36 grape samples and 42 wines from the three areas of Hexi Corridor region, namely, Jiayuguan, Zhangye and Wuwei, are subjects to be tested using the method established. Results show that mycotoxins, such as PCA, FB1, MPA, ZEN and CPA, can be detected in the grapes, among which MPA has the highest content (81.26 μg/L). Apart from the common OTA that could be detected in the wine, AFB2, CPA, ZEN and MPA are also found. The detection frequency of MPA is as high as 48%. MPA, CPA and ZEN could all be detected in the grapes and wines. Although there is no limit standard for these toxins in wine, their contents do not exceed the maximum limits in other food. That is to say, the pollution risks of mycotoxins to grapes and wines in Hexi Corridor region are relatively low. However, the routine monitorings to mycotoxins are still necessary. In addition, whether the mycotoxins in wine are brought from grape materials or directly produced by fungi remains to be further explored.

## Figures and Tables

**Table 1:** MS/MS acquisition parameters for mycotoxins.

Mycotoxin	Structure	Adduct ion	Precursor Ion (*m/z*)	Product ion *^a^* (*m/z*)	Fragmentor (V)	Collision Energy (V)
AFB1 (aflatoxin B1)	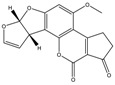	[M + H]^+^	313.0	**285.2**/241.1	130	**20**/20
AFB2 (aflatoxin B2)	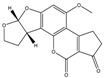	[M + H]^+^	315.1	**287.1**/269.1	130	**30**/30
AFG1 (aflatoxin G1)	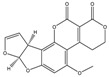	[M + H]^+^	329.1	**243.1**/311.1	130	**25**/20
AFG2 (aflatoxin G2)	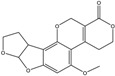	[M + H]^+^	331.1	**245.1**/285.1	130	**30**/25
AFM1 (aflatoxin M1)	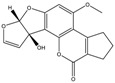	[M + H]^+^	328.9	**273.0**/228.9	165	**25**/50
CPA (cyclopiazonic acid)	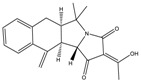	[M + H]^+^	337.3	**196.2**/182.1	39	**23**/20
CTN (citrinin)	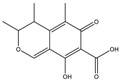	[M + H]^+^	250.9	**233.0**/205.0	80	**15**/25
DAS (diacetoxyscirpenol)	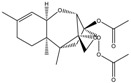	[M + NH_4_]^+^	384.0	**307.0**/247.0	65	**8**/10
DON (deoxynivalenol)	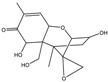	[M + H]^+^	297.2	**281.0**/265.0	120	**5**/25
FB1 (fumonisin B1)	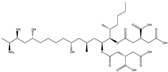	[M + H]^+^	722.1	**334.0**/352.1	100	**40**/35
HT-2 (HT-2 toxin)	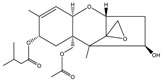	[M + Na]^+^	447.3	**284.8**/345.3	135	**20**/20
MEO (mevinolin)	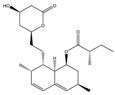	[M + H]^+^	405.1	**199.1**/285.1	125	**5**/5
MPA (mycophenolic acid)	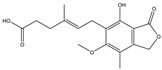	[M + H]^+^	321.1	**206.9**/274.7	90	**15**/15
NEO (neosolaniol)	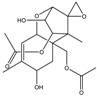	[M + NH_4_]^+^	400.0	**305.0**/185.0	95	**10**/15
OTA (ochratoxin A)	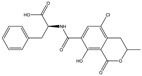	[M + H]^+^	404.0	**238.9**/357.9	127	**20**/10
OTB (ochratoxin B)	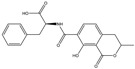	[M + H]^+^	369.9	**205.0**/324.0	115	**20**/10
PCA (penicillic acid)	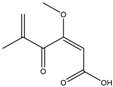	[M + H]^+^	170.8	**153.0**/125.0	10	**0**/15
STE (sterigmatocystine)	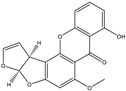	[M + H]^+^	325.0	**280.9**/252.9	135	**35**/55
T-2 (T-2 toxin)	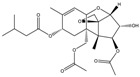	[M + NH_4_]^+^	484.1	**305.0**/215.0	115	**10**/18
ZEN (zearalenone)	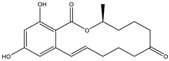	[M + H]^+^	319.0	**187.1**/184.9	70	**25**/40

*^a^* Quantifying ions indicated in bold.

**Table 2:** Matrix effect and recovery percentage obtained by applying various wine/water ratios (*v/v*).

Mycotoxin	Matrix Effect (%)/Recovery (%) (*n* = 3)					
	1:9	2:8	3:7	5:5	7:3	8:2
AFB1	−26/94	31/116	33/101	68/84	51/69	68/64
AFB2	−32/183	15/96	9/98	34/85	15/68	32/66
AFG1	−27/193	13/86	16/69	47/81	31/76	44/76
AFG2	−12/182	26/92	25/81	42/89	13/50	26/49
AFM1	−58/63	−60/72	−57/71	−64/84	−68/71	−69/76
CPA	−13/124	−27/88	−24/81	−37/88	−33/79	−30/63
CTN	53/84	37/88	50/89	54/94	61/88	68/85
DAS	312/84	289/90	325/90	357/96	413/88	435/89
DON	177/73	6/36	9/72	−21/115	−33/148	−18/240
FB1	−93/109	−92/94	−94/108	−93/101	−93/91	−92/74
HT-2	30/95	7/110	19/102	48/88	28/81	22/116
MEO	113/62	97/61	67/73	89/84	0/85	7/77
MPA	−23/116	−7/90	−24/107	8/81	−11/88	19/68
NEO	−8/71	−43/90	−41/74	−51/86	−57/80	−56/75
OTA	−14/120	1/106	−31/126	−1/106	−16/111	29/74
OTB	76/87	64/90	84/85	81/91	85/87	92/84
PCA	−48/105	−45/90	−59/115	−43/83	−61/102	−46/70
STE	−33/196	−7/145	−16/175	31/115	11/116	51/92
T-2	556/78	455/100	508/95	496/105	532/94	541/94
ZEN	−25/104	−14/98	−27/115	3/89	−11/79	12/67

**Table 3:** Matrix effect and recovery percentage obtained by applying various amounts of salt.

Mycotoxin	Matrix Effect (%)/Recovery (%) (*n* = 3)							
	A *^a^*	B	C	D	E	F	G	H
AFB1	−26/100	31/118	33/89	68/100	51/89	68/87	53/107	55/89
AFB2	−32/120	15/103	9/113	34/104	15/124	32/74	11/99	31/87
AFG1	−27/113	13/109	16/108	47/101	31/102	44/84	33/104	37/88
AFG2	−12/117	26/115	25/117	42/117	13/112	26/100	23/108	22/85
AFM1	−71/46	−72/56	−73/68	−72/65	−73/83	−73/79	−71/80	−71/88
CPA	−26/52	−26/78	−33/92	−32/94	−21/98	−32/116	−30/109	−17/94
CTN	46/55	52/64	53/69	47/78	48/93	46/93	47/87	40/93
DAS	118/68	176/72	187/82	198/85	221/93	209/92	209/89	198/92
DON	−59/158	−74/257	−59/120	−62/134	−67/95	−75/80	−39/77	−46/84
FB1	−94/100	−93/99	−93/98	−92/86	−94/85	−94/134	−94/74	−95/101
HT-2	31/98	15/102	44/96	33/94	33/103	36/90	46/89	21/100
MEO	−15/89	−33/93	−6/102	−26/117	−20/123	−21/113	−32/88	−21/90
MPA	−35/66	−31/66	−39/73	−40/86	−32/92	−39/97	−35/105	−24/94
NEO	−53/54	−53/63	−53/73	−51/72	−49/85	−58/89	−51/85	−53/91
OTA	−29/67	−20/57	−28/65	−33/77	−27/80	−35/87	−5/97	3/92
OTB	74/57	80/66	79/73	75/79	76/86	78/83	76/90	77/90
PCA	−52/76	−58/80	−64/77	−69/127	−68/103	−72/96	−14/110	6/89
STE	−33/59	−31/59	−29/58	−26/65	−15/68	−21/70	−50/112	−44/102
T-2	370/43	375/57	359/69	307/85	297/104	283/103	278/95	291/93
ZEN	−31/62	−33/64	−39/69	−38/79	−35/88	−38/89	−46/100	−40/95

*^a^* A (1.0 g MgSO_4_); B (2.0 g MgSO_4_); C (3.0 g MgSO_4_); D (4.0 g MgSO_4_); E (1.0 g NaCl + 1.0 g MgSO_4_); F (1.0 g NaCl + 2.0 g MgSO_4_); G (1.0 g NaCl + 3.0 g MgSO_4_); H (1.0 g NaCl + 4.0 g MgSO_4_).

**Table 4:** Linear regression data, LODs and LOQs for 20 mycotoxins analyzed.

Mycotoxin	Calibration	*R* ^2^	Linear Range (μg/L)	LOD (μg/L)	LOQ (μg/L)
AFB1	y = 5.6980x + 0.1230	0.9990	5–200	0.25	0.75
AFB2	y = 33.0713x + 116.2999	0.9993	1.25–50	0.13	0.39
AFG1	y = 71.1585x + 249.3916	0.9988	5–200	0.25	0.75
AFG2	y = 10.0093x + 4.5423	0.9991	1.25–50	0.06	0.18
AFM1	y = 4.4951x + 3.7299	0.9995	5–200	0.1	0.3
CPA	y = 3.1361x − 13.8214	0.9987	5–200	0.25	0.75
CTN	y = 923.0092x + 1433.5419	0.9995	5–200	0.1	0.3
DAS	y = 0.8656x − 1.8377	0.9980	5–200	0.1	0.3
DON	y = 0.8969x + 3045.7580	0.9971	5–200	5	15
FB1	y = 0.4647x − 0.9568	0.9994	5–200	1	3
HT-2	y = 0.0326x + 0.1858	0.9963	5–200	10	30
MEO	y = 1.6624x − 4.5656	0.9992	5–200	0.1	0.3
MPA	y = 6.9560x − 16.4431	0.9993	5–200	0.1	0.3
NEO	y = 42.1866x + 72.9181	0.9994	5–200	0.1	0.3
OTA	y = 3.6812x − 6.8163	0.9998	5–200	0.1	0.3
OTB	y = 195.8790x + 507.8370	0.9991	5–200	0.1	0.3
PCA	y = 10.5717x + 34.0765	0.9993	5–200	1	3
STE	y = 5.9077x − 0.9029	0.9999	5–200	0.1	0.3
T-2	y = 1.0423x − 2.7770	0.9983	5–200	0.25	0.75
ZEN	y = 0.5345x − 0.5351	0.9992	5–200	1	1

**Table 5:** Contamination levels of investigated mycotoxins in test grape samples with their frequency of occurrence and concentration ranges (μg/L).

Area	Total Number of Samples	Number of Samples with Mycotoxins Detected				
		PCA *^a^*	MPA	CPA	FB1	ZEN
Jiayuguan	3	-	1 (9.27)	3 (2.94–7.44)	-	-
Zhangye	9	9.04	2 (0.10–0.68)	-	-	-
Wuwei	24	11.71	2 (0.10–81.26)	5 (0.11–0.17)	1 (0.84)	2 (0.29–0.36)

***^a^*** - means not detected.

**Table 6:** Contamination levels of investigated mycotoxins in test wine samples with their frequency of occurrence and concentration ranges (μg/L).

Year	Total Number of Samples	Number of Samples with Mycotoxins Detected				
		OTA *^a^*	MPA	CPA	AFB2	ZEN
2007	1	-	1 (1.37)	-	-	-
2008	1	-	-	-	-	-
2009	2	-	2 (1.37–1.53)	-	-	-
2010	2	-	1 (1.53)	-	1 (0.10)	-
2011	2	-	1 (1.45)	-	-	-
2012	2	-	2 (1.60)	-	-	-
2013	3	-	2 (1.45–1.60)	1 (3.72)	-	-
2014	9	1 (1.27)	6 (1.45–2.31)	1 (3.90)	1 (0.10)	2 (1.82–1.85)
2015	14	1 (1.27)	5 (1.37–2.31)	3 (2.71–4.62)	-	1 (1.82)
2016	6	-	-	-	-	-

***^a^*** - means not detected.

## Supplementary Materials

The following are available online. Figure S1: Geographic sketch map of Hexi Corridor, Table S1: The basic meteorological data of the Hexi Corridor region (according to statistics from 1981 to 2010), Table S2: Matrix effect and recovery percentage obtained by applying various dSPE clean-up sorbents and their mixtures, Table S3: Values of matrix effect observed at three concentration levels, Table S4: Recoveries and precisions for 20 mycotoxins which were spiked at three levels.
