# Supplementary material for: Simultaneous Analysis of 20 Mycotoxins in Grapes and Wines from Hexi Corridor Region (China): Based on a QuEChERS–UHPLC–MS/MS Method
Source: Molecules. 2018 Aug 2;23(8):1926. doi: 10.3390/molecules23081926 (PMC6222325; doi:10.3390/molecules23081926)
Supplement: Supplementary file 1 [file molecules-23-01926-s001.pdf]

Supplementary Material:

Simultaneous analysis of 20 mycotoxins in grapes and wines from Hexi Corridor region (China): Based on a QuEChERS-UHPLC-MS/MS method

Bo Zhang†, Xia Chen†, Shun-Yu Han*, Min Li, Teng-Zhen Ma, Wen-Jun Sheng and Xia Zhu

Gansu Key Laboratory of Viticulture and Enology, College of Food Science and Engineering, Gansu Agricultural University, Lanzhou 730070, China.

† Both authors contributed equally to this work.

* Corresponding author.

Address: No. 1, Yingmen Town, Anning District, Lanzhou City, Gansu Province, 730070, PR China.

Tel.: +86-0931-7632-968.

Fax: +86-0931-7631-201.

E-mail: hanshunyu@gsau.edu.cn.

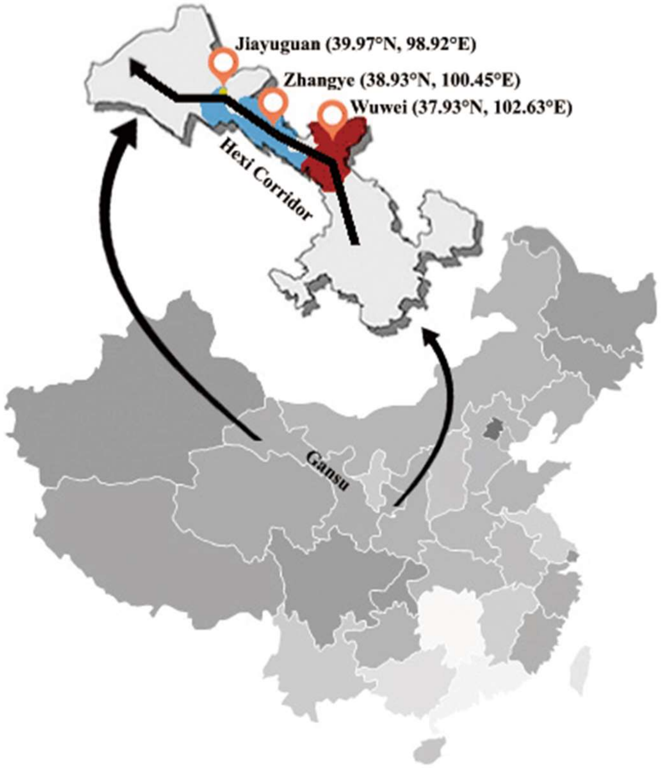

Figure S1. Geographic sketch map of Hexi Corridor.

Table S1. The basic meteorological data of the Hexi Corridor region (according to statistics from 1981 to 2010 [a]).

Month	Area	Average temperature (°C)	Average maximum temperature (°C)	Extreme maximum temperature (°C)	Average minimum temperature (°C)	Extreme minimum temperature (°C)	Average relative humidity (%)	Average precipitation of 20:00-20:00 (mm)	Average precipitation of 08:00-08:00 (mm)	Average wind speed (m/s)
January	Wuwei	-7.2	0.3	15.5	-13.3	-25.3	49.0	1.6	1.6	1.4
	Zhangye	-9.1	0.2	18.4	-15.9	-28.1	54.0	2.1	2.1	1.5
	Jiayuguan	-8.9	-1.6	13.0	-14.8	-27.8	55.0	1.5	1.5	1.9
February	Wuwei	-3.1	4.1	22.3	-9.3	-25.0	44.0	2.3	2.4	1.6
	Zhangye	-4.4	4.3	24.2	-11.4	-27.5	45.0	1.4	1.4	1.8
	Jiayuguan	-4.4	2.9	16.1	-10.6	-24.6	46.0	1.3	1.3	2.1
March	Wuwei	3.3	10.3	28.1	-2.9	-19.3	44.0	7.2	6.8	1.9
	Zhangye	2.6	10.7	26.2	-4.2	-18.8	43.0	4.1	4.0	2.2
	Jiayuguan	2.1	9.5	25.2	-3.9	-25.7	42.0	6.1	5.9	2.4
April	Wuwei	10.9	18.2	32.7	3.7	-7.1	38.0	8.3	8.5	2.3
	Zhangye	10.4	18.3	33.3	2.8	-8.0	38.0	5.3	5.3	2.5
	Jiayuguan	10.1	17.7	31.7	3.2	-9.8	34.0	3.3	3.5	2.9
May	Wuwei	16.2	23.2	34.1	8.6	-2.8	45.0	18.0	18.0	2.0
	Zhangye	16.2	23.8	34.7	8.3	-4.5	43.0	14.1	14.0	2.2
	Jiayuguan	16.2	23.4	33.4	8.8	-3.4	37.0	8.4	7.8	2.4
June	Wuwei	20.1	27.1	35.0	12.4	2.8	52.0	28.2	27.9	1.8
	Zhangye	20.3	27.9	36.7	12.6	4.1	49.0	20.8	20.5	1.9
	Jiayuguan	20.6	27.4	34.6	13.3	5.1	45.0	14.0	14.3	2.1
July	Wuwei	22.2	29.3	40.8	14.9	7.6	56.0	30.2	30.2	1.7
	Zhangye	22.3	30.0	39.8	15.1	6.7	54.0	28.7	29.0	1.9
	Jiayuguan	22.3	29.3	38.1	15.2	8.4	52.0	18.8	18.4	2.0

Table S1. *Cont.*

Month	Area	Average temperature (°C)	Average maximum temperature (°C)	Extreme maximum temperature (°C)	Average minimum temperature (°C)	Extreme minimum temperature (°C)	Average relative humidity (%)	Average precipitation of 20:00-20:00 (mm)	Average precipitation of 08:00-08:00 (mm)	Average wind speed (m/s)
August	Wuwei	20.7	27.8	36.5	14.0	4.3	59.0	35.4	35.2	1.6
	Zhangye	20.7	28.5	37.5	13.7	4.5	57.0	27.2	27.1	1.8
	Jiayuguan	20.6	28.1	35.2	13.4	4.4	52.0	17.1	17.6	1.9
September	Wuwei	15.4	22.6	34.9	9.3	-0.8	62.0	24.9	25.2	1.4
	Zhangye	15.0	23.5	34.5	8.4	-1.1	61.0	19.6	19.6	1.6
	Jiayuguan	14.9	22.8	33.0	8.3	-3.7	53.0	10.9	10.7	1.8
October	Wuwei	8.5	16.2	27.8	2.3	-14.4	58.0	10.8	11.1	1.4
	Zhangye	7.3	16.5	30.3	0.7	-12.7	59.0	5.8	6.0	1.5
	Jiayuguan	7.6	15.5	26.7	1.5	-16.9	49.0	3.5	3.9	1.9
November	Wuwei	0.8	8.6	22.8	-5.0	-20.5	54.0	2.7	2.7	1.4
	Zhangye	-0.6	8.4	22.3	-6.5	-19.3	58.0	1.8	1.9	1.7
	Jiayuguan	-0.3	7.0	19.4	-5.7	-20.6	51.0	2.0	2.0	2.0
December	Wuwei	-5.4	1.9	17.9	-10.9	-32.0	54.0	1.5	1.5	1.4
	Zhangye	-7.3	1.7	19.6	-13.4	-28.2	58.0	1.7	1.7	1.6
	Jiayuguan	-7.3	-0.3	12.9	-12.7	-29.8	57.0	1.5	1.5	1.8

[a] The data come from National Meteorological Information Center of China.

Table S2. Matrix effect and recovery percentage obtained by applying various dSPE clean-up sorbents and their mixtures.

Mycotoxin	Matrix effect (%)/recovery (%) (*n*=3)						
	**PSA**	**C18**	**GCB**	**C18+GCB**	**PSA+GCB**	**C18+PSA**	**No clean-up**
AFB1	33/96	-44/85	-21/92	-31/23	28/77	14/84	-24/82
AFB2	26/107	-45/89	-19/71	-20/62	34/66	-34/79	-34/82
AFG1	35/92	-34/103	-33/81	-31/54	6/65	11/81	-15/78
AFG2	12/88	24/111	-10/13	3/69	-10/81	-31/89	-21/84
AFM1	-83/70	-79/85	-77/18	-79/16	-79/16	-79/73	-81/84
CPA	7/95	17/98	13/66	34/53	32/46	31/81	17/97
CTN	52/72	64/80	71/46	66/39	63/42	57/80	64/81
DAS	10/73	49/121	53/81	45/90	45/90	39/56	63/79
DON	-27/81	-44/67	-67/70	-77/104	-69/59	-78/76	-56/66
FB1	-54/1	-62/90	-64/71	-62/60	-74/1	-67/1	-69/106
HT-2	-10/95	12/118	5/86	0/95	7/100	7/66	24/87
MEO	121/106	-23/113	-66/66	-37/77	167/78	103/81	179/90
MPA	-35/122	22/88	14/78	36/65	27/66	30/94	14/100
NEO	-49/73	-2/88	-32/71	-1/78	-36/73	15/57	-47/76
OTA	-4/95	113/100	116/23	162/18	134/11	132/62	110/104
OTB	78/34	95/98	107/44	98/39	104/13	90/79	100/79
PCA	-9/115	2/95	11/84	34/67	11/92	17/101	-10/102
STE	-20/127	-48/103	-45/3	-16/2	-17/1	-4/94	-24/88
T-2	22/68	27/81	26/71	20/78	27/85	15/79	20/88
ZEN	-40/120	-33/89	-36/78	-19/61	-22/59	-17/92	-33/93

Table S3. Values of matrix effect observed at three concentration levels.

Mycotoxin	Matrix effect (%) [a]		
	**Level 1**	**Level 2**	**Level 3**
AFB1	-41.59	12.50	-22.74
AFB2	88.44	-19.75	-63.57
AFG1	43.96	52.17	-33.05
AFG2	17.39	74.19	-33.03
AFM1	19.35	41.90	-39.11
CPA	5.66	135.35	-10.18
CTN	9.80	14.67	-6.59
DAS	27.36	61.79	3.77
DON	61.54	32.91	-25.47
FB1	-8.86	61.80	-44.28
HT-2	-18.56	-22.43	-10.00
MEO	44.12	80.43	-21.17
MPA	-0.54	11.34	-2.71
NEO	70.37	43.75	61.67
OTA	48.33	107.89	-33.24
OTB	16.86	116.09	-32.58
PCA	-37.19	-11.42	20.95
STE	6.62	27.08	-14.02
T-2	24.61	25.92	-21.28
ZEN	4.88	48.28	-0.89

[a] Level 1, Level 2, Level 3 were the concentration 2.5 μg/L, 5 μg/L and 10 μg/L, respectively.

Table S4. Recoveries and precisions for 20 mycotoxins which were spiked at three levels.

Mycotoxin	Recovery (%) [a]			Average recovery (%)	Precision RSD (%) (*n*=6)
	Level 1	Level 2	Level 3		
AFB1	90.91	95.45	114.52	100.29	10.23
AFB2	103.61	94.77	84.85	94.41	7.66
AFG1	75.32	78.83	109.71	87.95	15.45
AFG2	96.30	72.22	114.66	94.39	17.38
AFM1	86.49	85.23	112.48	94.73	12.56
CPA	94.23	105.86	107.98	102.69	6.05
CTN	91.07	87.60	102.46	93.71	6.35
DAS	84.63	81.46	115.05	93.71	15.14
DON	87.62	71.43	102.60	87.22	12.73
FB1	112.50	114.58	126.38	117.82	6.11
HT-2	88.24	115.38	100.00	101.21	11.12
MEO	81.12	89.92	121.84	97.63	17.49
MPA	91.26	104.94	95.93	97.38	5.68
NEO	84.78	77.78	94.27	85.61	6.76
OTA	79.40	90.51	118.28	96.06	16.36
OTB	89.98	90.61	121.50	100.69	14.71
PCA	86.55	110.76	81.83	93.05	12.67
STE	82.84	81.48	105.47	89.93	11.00
T-2	83.20	82.94	103.37	89.84	9.57
ZEN	88.37	102.78	93.69	94.95	5.95

[a] Level 1, Level 2, Level 3 were the concentration 2.5 μg/L, 5 μg/L and 10 μg/L, respectively.
